# Soluble urokinase plasminogen activator receptor (suPAR) in the emergency department: An update

**DOI:** 10.22088/cjim.13.4.650

**Published:** 2022

**Authors:** Dimitrios Velissaris, Nicholas Zareifopoulos, Vasileios Karamouzos, Charalampos Pierrakos, Menelaos Karanikolas

**Affiliations:** 1 Department of Internal Medicine, University Hospital of Patras, Patras, Greece; 2 Intensive Care Unit, University Hospital of Patras, Patras, Greece; 3 Department of Intensive Care, Brugmann University Hospital, Université Libre de Bruxelles, Brussels, Belgium; 4 Department of Anesthesiology, Washington University School of Medicine, St. Louis, MO, USA

**Keywords:** suPAR, Biomarker, Emergency department, Mortality, Infection, Sepsis

## Abstract

**Background:**

The biomarker soluble urokinase plasminogen activator receptor (suPAR) is an indicator of inflammation which is increased in a variety of chronic and acute disease states. Its most promising application in the emergency setting is to aid in the prognostic stratification of patients by identifying those at high risk of deterioration. This is a narrative review of studies evaluating the use of suPAR.

**Methods:**

We conducted a Medline search for studies on the use of suPAR in patients acutely admitted to the emergency department.

**Results:**

25 original studies were included in the review. suPAR as a marker of inflammation has been used alone or combined to other inflammatory biomarkers in the assessment of patients suffering from various acute and chronic diseases in an emergency setting. As it is non-specific, it may increase in infectious disease, malignancy or acute coronary syndromes among other conditions, but quantitative suPAR levels correlate with disease severity. It may be useful for the identification of high risk patients regardless of underlying pathology.

**Conclusion:**

As the ideal biomarker in the emergency setting has not been identified yet, suPAR may be a promising addition to the established biomarkers for the initial assessment of patients in this setting. Additional research is necessary to evaluate the usefulness of suPAR guided management algorithms.

Several biomarkers involved in different biological pathways have been used in the emergency department setting aiming to help clinicians in the diagnosis, risk stratification and monitoring of diseases. Concentration of the soluble urokinase plasminogen activator receptor (suPAR) in serum is intimately related to the immune and inflammatory status of the patients, and has been used in the recent years in the assessment of several diseases with multiple underlying pathophysiological processes (1, 2). The precursor protein of suPAR is expressed in various immune system cells and suPAR is released into the systemic circulation upon the activation of these cells. It is considered a non-specific biomarker as elevated plasma levels are encountered in a variety of both acute and chronic diseases apart from infection and sepsis, including malignant tumors, congestive heart failure and various autoimmune conditions (3). suPAR is a biomarker reflecting a low-grade inflammation, a mechanism that is present in the development of several diseases, such as infectious, cardiovascular, malignant diseases and more. Plasma levels of suPAR are also associated with social habits, such as smoking, alcohol consumption, and lifestyle (1, 4). The suPAR level is indicative of immune system activity and inflammatory processes.

Although suPAR does not belong to the common used tools of investigation in daily practice, meets some basic criteria of a useful biomarker because it reflects various underlying pathophysiology, remains stable in plasma and is not significantly affected by the circadian cycle. Normal suPAR level in healthy individuals’ plasma is below <3 ng/ml, in unselected patients in the emergency department 3-6 ng/ml, and in critically ill patients is > 6 ng/ml (2, 5, 6). Due to its lack of specificity, it is not particularly useful for diagnostic purposes, although low levels can be used in combination with other biomarkers to rule out possible causes for patients’ complaints. Its most promising application is in the prognostic stratification of patients presenting to different medical settings, as measurements seem to correlate with disease severity and mortality. Previous research has focused on the use of suPAR as a prognostic marker in the inpatient, outpatient and intensive care setting where it proves to be predictive of mortality but the information it offers does not appear to influence clinical practice. It is predictive of mortality in acute coronary syndromes and marked suPAR elevation is commonly observed in septic shock. The purpose of this study is to evaluate the clinical value of suPAR in the emergency department setting, where clinicians are required to decide upon a course of action immediately while the underlying cause of the patients’ complaints is initially unclear. Our hypothesis is that suPAR as a biomarker may be valuable for the early identification of patients with severe illness who require intensive care, regardless of the actual diagnosis. As it is not specific for a particular disease, we expect its value as an aid to the diagnostic process to be limited.

The identification of a biomarker with the highest validity for diagnosis, prognosis and management of patients in the emergency department remains of high priority. This is an update of the current literature regarding the role of suPAR as a diagnostic and prognostic biomarker when used in the setting of an acute care ward. 

## Methods

**Figure 1:**
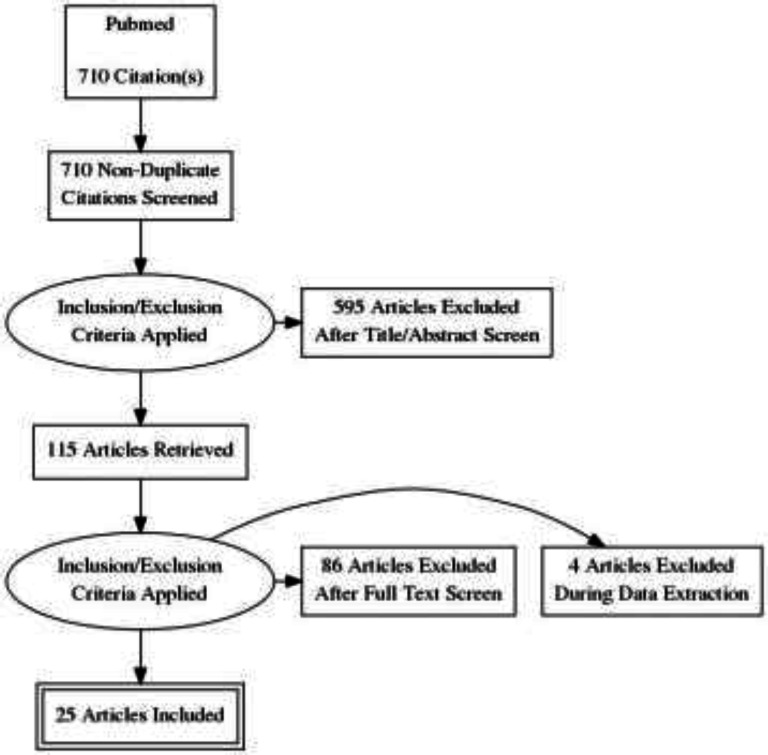
PRISMA flow chart of the literature review

A Medline search was conducted and the search terms were ‘suPAR’ and ‘Emergency Department’. After the selection of the most suitable articles to the research object, the bibliographies were reviewed, and additional relevant publications were extracted. We intended to include the use of suPAR as a prognostic indicator of patients admitted to the ED in the review cohort studies, regardless of the underlying diagnosis. Studies with a sample of at least 10 patients with initial evaluation in the emergency department and subsequent follow-up were to be included, likewise the provided relevant data to the use of suPAR as a prognostic indicator. Articles not relevant to the emergency setting, articles focusing exclusively on COVID-19 and articles not available in English were excluded. Though this was not a systematic review, a PRISMA flow chart is provided for the study (figure 1). The results were current as of December 20, 2020. Institutional ethics review board approval was waived for this work as it did not involve human or animal subjects. The work is compliant with ethical standards as dictated by the 1975 declaration of Helsinki.

## Results

The articles retrieved during the review process are presented in table 1 chronologically. The study population, main findings and conclusions of each study are listed, together with any additional notable comments. The role of suPAR as a prognostic and diagnostic biomarker has been investigated in large cohorts of patients admitted to the ED. In one article published in 2012, suPAR elevation was identified as an independent negative prognostic factor in patients with a variety of ailments (7). In 2016, in a retrospective study 4, 343 patients were enrolled and a strong correlation was found between elevated suPAR values and adverse outcomes, most notably mortality and subsequent ED admissions. suPAR measurements may provide important insight into disease prognosis, as adverse events were much more common in groups of patients with elevated suPAR on admission and conversely those with lower initial values were less likely to die or readmitted to the ED (8). 

A study by the same group in 2018 consecutively enrolled 17, 312 admitted patients with the intention to investigate whether the prognostic accuracy of the National Early Warning Score (NEWS) could be improved by the addition of suPAR elevation to the scoring system. In this large cohort of patients, the suPAR-NEWS composite score could identify high and low risk groups of patients more accurately the NEWS score when used alone. Intriguingly, elevated suPAR measurements were associated with increased mortality even in patients for whom the NEWS score alone indicated a minimal risk of deterioration (9). 

The interventional prospective trial (TRIAGE III) consecutively included 16,801 patients admitted to the ED to evaluate whether a triage system guided by suPAR measurements could affect mortality in the trial centers. Implementation of this system had no effect of mortality. The association between suPAR elevation and mortality was however noted in this cohort as well at multiple follow-up time points (10). Based on this trial, two further articles led to some more useful conclusions regarding suPAR. The first one, a post-hoc analysis of TRIAGE III trial indicated that suPAR measurements could more accurately discern a 7-day mortality risk when combined with routine triage processes (11). And the second one, another post-hoc sub-study, demonstrated that readily available suPAR measurements in the ED resulted in increased 24-hour discharge rates and shorter hospital stays at the cost of a higher rate of readmissions (12). 

This is in contrast with the findings of a previous study, which demonstrated that using suPAR to guide management in the ED did not lead to increased short-term readmissions (within the first few days), but was associated with a greater risk of readmission within a month after discharge (13). A pilot study regarding the use of suPAR for rapid prognostic stratification and triage in a resource limited setting showed promising results, but further data is warranted before suPAR guided algorithms could be incorporated into clinical practice (14). With regard to the assessment of SIRS patients in the ED, a study showed that among several studied biomarkers, suPAR was not superior to others (15). To the contrary of this study, the other ones indicated that suPAR could contribute to the prediction of bacteremia in SIRS patients (16), was found suitable to differentiate SIRS patients with and without positive blood cultures (17), was found the most promising biomarker among evaluation of nine biomarkers in early SIRS (18) and in the cases of early SIRS, suPAR plasma level was found predictive for mortality (19). 

Furthermore, a study showed that elevated suPAR level predicts case fatality and severe sepsis in patients with suspected infection (20) and in a three –center Italian study, lactate and suPAR were the most accurate predictors of adverse outcomes for patients admitted to the emergency ward on suspicion of infection (21). Recently it has been suggested that there may be a correlation between suPAR elevation on ED admission and risk for both acute kidney injury during hospitalization and subsequent development of chronic renal failure (22). A large multicenter study suggested that suPAR may be predictive of complications of sepsis that may arise as a result of endothelial stress, including septic shock, renal failure and hepatic failure (23).

The role of suPAR has been examined in specific clinical conditions. In the exacerbation of chronic obstructive pulmonary disease (AECOPD), it was found that monitoring serum suPAR could be helpful in the assessment of treatment response and in the determination of the AECOPD prognosis (24). In cases of acute pancreatitis, had a significant value in indicating the severity of the acute disease (25) and in a retrospective study suPAR elevation was associated with an increased risk of emergency surgery and greater postoperative mortality during the first 3 months of follow- up (26). In regard to cardiac diseases, a study showed that suPAR could reliable predict mortality in patients with suspected acute myocardial infarction in the ED (27), but another article by the same group showed that circulating levels of suPAR on top of high sensitive troponin I (hs-TnI) do not improve the early diagnosis of AMI (28). This was also assessed in an article published in 2013, in which suPAR was used for prognostic stratification of patients presenting to the ED with acute chest pain and increased levels were associated with mortality (29). 

Another notable cohort study included 22653 patients between 40 and 69 years old and 19889 individuals over the age of 70. Its primary purpose was to examine the validity of prognostic stratification models across a relatively wide age range. A suPAR measurement was available for 6400 individuals, demonstrating that it retained a relatively higher prognostic validity in middle age and younger patients compared with the geriatric subgroup (30). In another study that acutely admitted patients >65 years were assessed and results showed that suPAR measurements may have significant associations with organ dysfunction and physical performance status (31). Another prospective multi-center study of 136 geriatric individuals over age 75 who received emergency treatment for infection concluded that among several biomarkers (including suPAR), MR-proADM was the most reliable and accurate predictor of 30-day mortality (32). 

**Table 1:** Synopsis of studies on the use of suPAR in the emergency setting

Author, year of publication	Study design	Study population	Aim of the study	Major findings	Conclusions
Kofoed, 2007	Prospective cohort study	151 eligible patients, 96 with bacterial infection	Comparison of the diagnostic performance of suPAR, sTREM-1, MIF, PCT, CRP	suPAR AUC (0.4-0.6) for the diagnosis of bacterial infection when used alone. The composite index including all measured biomarkers was superior to any biomarker used alone	suPAR was of limited specificity for the diagnosis of bacterial infections and inferior to both procalcitonin and CRP.
Haupt, 2012	Prospective observational study	543 patients admitted to an emergency department in Denmark during a 2-month period.	Evaluation of the prognostic significance of increased serum suPAR in combination with the Charlson score	suPAR elevation correlated well with the Charlson score and was an independent predictor of mortality and increased duration of inpatient treatment, but not of readmission. Higher suPAR levels were observed in patients with a wide variety of diseases, specifically malignancies, hepatic disease and coronary heart disease.	SuPAR may be a useful independent prognostic biomarker in an emergency setting, aiding in accurate risk stratification of patients suffering from a wide range of ailments.
Uusitalo-Seppälä 2012	Prospective single-center cohort study	539 individuals admitted to an emergency department on suspicion of infection	Evaluation of suPAR as a prognostic biomarker in acute bacterial infections.	A statistically significant difference (p<0.001) in average suPAR levels was observed in patients with severe sepsis and those who died compared to those with milder infection and survivors, respectively.	SuPAR elevation may be predictive of mortality and severe sepsis in the emergency setting.
Hoenigl, 2012	Comparative study	132 individuals admitted to an emergency ward with signs of systemic inflammatory response syndrome (SIRS).	The study was designed to compare the diagnostic and prognostic role of CRP, suPAR procalcitonin and Interleukin-6 in cases of SIRS	suPAR elevation was associated with higher mortality and sepsis confirmed by positive blood cultures. No significant prognostic implication of the other biomarkers was observed.	The use of suPAr alonside procalcitonin and interleukin-6 may aid in the timely identification of septic patients and in their progostic stratification.
Lyngbaek, 2013	Single center cohort study	449 consecutive chest pain patients.	To evaluate the prognostic value of suPAR in patients presenting with chest pain to the emergency department without evidence of ST- elevation myocardial infarction.	A significant and independent correlation was found between increased levels of suPAR and mortality during the follow-up period. Abnormal electrocardiographic findings and troponin measurements were also associated with suPAR elevation	Elevated suPAR levels may be predictive of adverse outcomes and mortality in patients presenting to the emergency department due to chest pain, regardless of the underlying diagnosis.
Loonen AJM, 2014	Retrospective cohort study	The study sample was comprised of 140 patients admitted to an emergency ward in the Netherlands with signs of SIRS and clinical evidence of infection.	Evaluation of the ability of CRP, PCT, suPAR, and neutrophil/lymphocyte ratio (NLCR) to predict blood stream infection and sepsis in an emergency setting.	Significantly higher levels of procalcitonin, suPAR and NLCR were observed in patients with positive blood cultures (p<0.01 for all biomarkers).	suPAR may be useful to differentiate septic patients from those with non-infectious SIRS. The studied molecular assays performed poorly in the assessed ED patients. The NCLR and procalcitonin may be of similar utility.
Raggam RB, 2014	Prospective cohort study	902 adult patients with SIRS	To assess the prognostic value of suPAR in early SIRS patients.	After multivariable regression analyses, suPAR concentration on admission was associated with increased mortality at 2, 30 and 90 days of follow-up.	Early suPAR elevation in SIRS may be a useful predictor of mortality.
Reichsoellner, 2014	Prospective cohort study	159 patients with SIRS	9 biomarkers of inflammation were evaluated in regard to their diagnostic and prognostic performances in SIRS patients.	Among the assessed biomarkers, the most accurate preditors of positive blood cultures and mortality at 30 days of follow-up were interleukin-8, biotin, suPAR and procalcitonin.	suPAR was identified as the most promising prognostic biomarker in patients with early SIRS.
Nayak RK, 2015	Prospective cohort study	1,036 patients were enrolled	To determine if suPAR was related to readmission and patients’ mortality in the acute medical setting.	The highest suPAR tertile level in analysis was significantly associated with mortality within 30 days after discharge. Also, a significant association was found with readmission within the maximum observation period of the patients.	Elevated suPAR levels are related to increased long-term readmission rates, but suPAR is not an independent biomarker for increased risk of short-term readmission in the acute medical setting.
Casagranda, 2015	Multi-center prospective trial in the EDs of 3 Italian hospitals	Patients with sepsis	Examination of the role of suPAR measurements in patients with sepsis in an emergency setting.	Lactate, suPAR and procalcitonin levels on admission were significantly higher in cases of severe sepsis and septic shock compared to milder cases. A tendency of suPAR levels to gradually decline during the course of hospitalization was observed. Baseline suPAR levels were independently associated with mortality at the 30-day follow-up point.	The most accurate predictor of 30-day mortality may be suPAR in patients admitted with sepsis, but lactate was a more accurate predictor of mortality at the 7-day follow-up point.
Rasmussen, 2016	Registry-based retrospective cohort study	4,343 consecutive patients presenting to the emergency ward of a hospital in Denmark.	The study was designed as to examine the prognostic value of suPAR in patients presenting to the emergency room regardless of the primary complaint.	An association was observed between increased suPAR levels and age, duration of hospital stay, risk of admission to the intensive care unit and risk of re-admission after discharge.	Elevated suPAR levels on admission may be associated with increased risk of adverse outcomes, whereas low baseline suPAR concentrations indicate a more favorable prognosis regardless of the underlying diagnosis.
Klausen HH, 2017	Single-center cross-sectional study	369 acutely admitted patients aged >65 years.	Aim of the study was to examine the association between inflammatory biomarkers (tumor necrosis factor-a, interleukin-6 and suPAR), performance status and risk of organ dysfunction in geriatric patients	The most accurate prognostic indicator of adverse outcomes and decreased performance status is suPAR in comparison with TNF-a and IL-6.	SuPAR may be a useful indicator of frailty and risk of deterioration in performance status in geriatric patients (age>65).
Rasmussen, 2018	Registry based observational cohort study	17,312 consecutively admitted acute patients	To investigate if suPAR measurements provide additional prognostic value in combination with the National Early Warning Score-NEWS score in an emergency care setting.	Elevated suPAR on admission was associated with increased mortality at 30 and 90 days of follow-up independently of the NEWS score. A composite index combining NEWS, age, and sex with suPAR improved prediction of mortality at all follow-up points.	High baseline suPAR levels may be associated with increased mortality regardless of the NEWS score. The subgroup of individuals with high suPAR and low NEWS score was characterized by similar mortality to the subgroup of patients with a high NEWS score.
Schultz, 2018	Prospective clinical trial (TRIAGE III)	16801 consecutive patients presenting to hospitals of Denmark capital region.	The study was designed to evaluate suPAR as a prognostic biomarker in the ED and the effects of utilizing suPAR based on clinical decision-making algorithms on patient outcomes.	A significant association was found between increased suPAR and mortality at the 30-day follow-up point. The introduction of suPAR measurements did not greatly influence clinical decision-making and effect on mortality was noted.	The availability of suPAR measurements did not exert any effect on the outcome of patients admitted to the emergency department.
Meyer, 2018	Retrospective registry-based cohort study	17312 individuals presenting to an emergency department in Denmark	To assess the role of suPAR in the prediction of acute surgery cases compared to elective and post-operative mortality.	Higher suPAR levels on admission were observed in patients who required emergency surgery as compared with those who required no surgery or were offered surgery on an elective basis.	Elevated suPAR levels on emergency department admission were associated with an increased risk of emergency surgery and postoperative mortality.
AboEl-Magd GH, 2018	Prospective cohort study	45 patients admitted for an acute exacerbation of chronic obstructive pulmonary disease and 20 healthy controls in an Egyptian hospital.	The study was designed to assess the potential role of suPAR in diagnosing COPD exacerbations and indicating response to treatment.	The group of patients with an acute COPD exacerbation had a significantly higher suPAR level at baseline compared to the control group. A trend towards declining suPAR levels was observed following initiation of treatment.	Monitoring serum suPAR may aid in the timely diagnosis and prognostic stratification of acute COPD exacerbations.
Küçükceran, 2018	Prospective cohort study	59 patients with pancreatitis	The study was intended to evaluate the prognostic value of suPAR in acute pancreatitis	Elevated suPAR levels were observed in severe and necrotic cases of acute pancreatitis as compared with milder cases.	suPAR had a significant value in indicating the severity of acute pancreatitis
Schultz, 2019	Post hoc analysis of Schultz 2018 (TRIAGE III)	4,420 participants of the TRIAGE III study	Evaluation of the utility of suPAR to aid triage and risk stratification and the effect on mortality	suPAR levels above 5.9 ng / ml were more accurate in predicting mortality than the previous clinical triage systems.	There may be value in using suPAR levels alongside conventional clinical triage methods to reclassify patient risk status.
Schultz, 2019	A sub-study of the interventional TRIAGE III trial	Post hoc sub-study, same population as TRIAGE III	To evaluate if the availability of suPAR could lead to earlier discharges in the ED setting.	The use of suPAR levels to guide admissions led to a higher proportion of patients being discharged within 24 hours of presentation but also significantly increased the readmission rate.	No difference was noted on mortality in patients discharged within 24 hours of admission based on suPAR levels below the cutoff point.
Schultz M, 2019	Prospective cohort study	22653 patients (age 40-69 years), and 19889 patients over 70	Evaluation of various risk stratification triage models including suPAR	Vital sign-based algorithms were more accurate in middle age patients compared to geriatric cases. The most accurate individual biomarker predictive of mortality was suPAR	The predictive value was lower in elderly individuals in comparison to middle-aged patients for all the investigated models. Modifications for age should always be considered in risk assessment models in the ED patients.
Julián-Jiménez A, 2019	Prospective, observational, multicenter, analytical study	136 patients over 75 years of age	To analyze and compare individual biomarkers including suPAR for the prognostic stratification of sepsis.	Among the assessed biomarkers (including suPAR), MR-proADM was the most accurate predictor of mortality at 30 days of follow-up. The mixed model (MR-proADM plus qSOFA≥2) was superior than either indexed used alone.	MR-proADM was superior than suPAR as a predictor of mortality in elderly patients with sepsis
Kumar, 2019	Pilot, observational study	190 patients with acute illness in the ED in India.	To assess the role of suPAR in a low resource, densely populated country, as a rapid test for triage and prognostication in the ED alongside the emergency severity index (ESI).	A cutoff of 5.5 ng/ml of suPAR correlated well with ESI scores of 3 and lower, signifying cases requiring admission.	suPAR can be effectively utilized in the ED triage purposes.
Sörensen NA, 2019	Single center prospective cohort study	1314 ED patients admitted on suspicion of myocardial infarction.	Evaluation of the predictive value of suPAR level for mortality after a year of follow- up.	Median suPAR levels were similar in patients with and without a myocardial infarction. Elevated suPAR was however associated with a higher mortality rate after one year of follow-up.	suPAR may be of use in predicting long term mortality in patients presenting to the emergency department on suspicion of acute coronary syndromes.
Sörensen NA, 2019	Prospective cohort study	1220 admitted on suspicion of myocardial infarction.	Evaluation of the diagnostic value of suPAR combined with troponin measurements in cases of acute coronary syndrome	suPAR was not accurate as a diagnostic biomarker of myocardial infarction when used on its own and did not improve the sensitivity of serial troponins.	Measurement of suPAR does not aid in the timely diagnosis of myocardial infarctions.
Lafon, 2020	Prospective, multicenter, international study conducted in 14 EDs	602462 patients with an acute community acquired bacterial infection	Evaluation of the role of endothelial biomarkers for predicting adverse outcomes of patients presenting to an emergency setting with signs of sepsis.	suPAR and procalcitonin were the most accurate biomarkers for predicting early complications in the clinical course of patients and clinical deterioration. A composite biomarker consisting of sVEGFR2 protein and suPAR was the most accurate predictor of adverse outcomes.	suPAR may be indicative of endothelial stress, a property underlying its prognostic accuracy for complications of sepsis such as organ dysfunction, diffuse intravascular coagulation and septic shock
Iversen, 2020	Retrospective registry-based cohort study	25497 patients admitted to the emergency department in 2 Danish hospitals	Evaluation of the suPAR levels for predicting the development of acute or chronic renal failure	High levels of suPAR on hospital admission predicted the development of acute kidney injury (Hazard ratio= 2.51 (95% CI: 2.09-3.01, P< 0.001) and chronic renal failure (Hazard ratio= 1.57 (95% CI: 1.38-1.78, P<0.001)	SuPAR measurements may aid in the identification of patients at risk for renal failure

## Discussion

A body of literature has been identified in regard to the use of soluble urokinase plasminogen activator receptor in the emergency department setting. Most of the related studies have been performed for the last 15 years, demonstrating the possible role of suPAR in the prognostic stratification of a broad spectrum of diseases in acutely admitted patients (5). SuPAR elevation in the emergency department has been associated with increased acute mortality and an increased risk of complications in patients with sepsis, non-infectious SIRS and acute coronary syndrome and may aid in the identification of high-risk patients when used in conjunction with other biomarkers and common clinical criteria. Though suPAR is used as a biomarker in a variety of settings in tertiary canters in Scandinavian countries, there are certain concerns that limit its widespread adoption: it is considerably more expensive than other common tests utilized in the emergency setting as it is performed by enzyme-linked immunosorbent assay (ELISA) and to this point no standard reference range for normal values has been established (1). It is exquisitely non-specific, a characteristic which may be considered beneficial as it can be used by a variety of different specialists for the evaluation of different disease processes. This does however limit its value for the initial diagnosis of patients presenting to the emergency department and the interpretation of suPAR levels in patients with multiple comorbid conditions which are expected to elevate them is quite complex, especially in the absence of an evidence-based reference range. There is insufficient evidence to recommend the routine use of suPAR in the evaluation of acute chest or abdominal pain. Preliminary findings suggest that suPAR elevation may be correlated with higher mortality in patients with acute coronary syndromes, but it does not offer any advantage compared to the use of serial electrocardiograms (ECGs) and troponin measurements (27). There is insufficient data regarding its utility in the evaluation of abdominal pain (9). It may be most useful in the initial evaluation of sepsis, as the extremely elevated levels of suPAR are specific for septic shock and may aid in identifying patients who require prompts treatment in an intensive care setting (4).

In the emergency department setting a proper risk assessment of patients is necessary to ensure that the most ill of them are prioritized, quickly examined and received the most careful observation. For that reason, triage systems are used for this risk assessment aiming to prioritize the order of patients to be treated. suPAR is a novel biomarker closely related to the underlying immune and inflammatory status of the patient. High suPAR levels are associated to the presence and progression of a disease and related to increased mortality risk. However, although suPAR seems to correlate well with other common used inflammatory biomarkers, it is not yet feasible to use suPAR guided algorithms to guide management of patients in the emergency ward. Discharging patients on the basis of low suPAR score at this time poses an unacceptable risk of emergent complications outside of the hospital and readmission with a worse prognosis than upon initial presentation (2). An extremely elevated level of SuPAR could however facilitate a decision to initiate intensive care for a patient with suspected sepsis, which may consist of administration of fluids at a faster rate, central venous catheter placement, administration of different antibiotics consideration of intubation and mechanical ventilation. Thus, it is clear that it may be useful for the identification of patients at high risk of sepsis complications but low levels should not be interpreted as implying minimal risk. In other medical conditions that might be evaluated in the ED such as acute coronary syndromes or pancreatitis, it is not yet clear whether the addition of suPAR to other ubiquitous laboratory tests would change clinical practice in a meaningful way. Since suPAR is elevated in chronic disease as well, it may be difficult to ascertain in patients with multiple comorbidities whether an elevated suPAR measurement in the emergency setting is associated with a condition of acute onset or if it reflects the chronic disease burden of the patient. This is especially true for individuals suffering from malignant or autoimmune diseases which are associated with significant suPAR elevation. This could limit the usefulness of suPAR in this high-risk group if baseline levels are not available (1, 2). An ideal biomarker would be expected to have near perfect sensitivity and specificity for the conditions it is used to evaluate, a quantitative correlation with disease severity and a measurement method which provides results in a timely manner which in the emergency room setting would be within a matter of minutes. No time lag would be expected to exist between the onset of the disease process and the elevation of the ideal biomarker levels above the diagnostic threshold. Based on the above description, the ideal biomarker does not exist for any condition, though troponin measurements for the evaluation of myocardial infarction are perhaps the test closest to this ideal. 

Articles published during the past decade are encouraging regarding the prognostic validity of suPAR measurements in an emergency setting. It is however far from the ideal biomarker and the evidence remains is not sufficient to recommend the widespread adoption of suPAR guided management algorithms. An additional drawback is the fact that outside the Scandinavian region is Europe clinical experience with this test is extremely limited.

There is no doubt that multiple benefits will arise when a biomarker that is closer to the ideal is discovered for acutely admitted patients. These would include the reduction of wait times in the emergency department, the reduction of the number of re-admissions to hospital and a better stratification for the management of the patients. suPAR is elevated in a number of several diseases and this characteristic may be useful for the clinicians, when using suPAR alone or in combination to other specific biomarkers of diseases in the attempt to increase diagnostic and prognostic accuracy. 

In conclusions suPAR may be useful in the assessment of ED patients admitted with various diseases in the rapid assessment, in the risk stratification and the determination of more intensive clinical assessment and monitoring care. Its prognostic and diagnostic validity needs further investigation with larger multicenter prospective cohort studies. As its value lies mostly in its correlation with adverse outcomes, further studies should focus on the use of suPAR in triage of patients presenting for emergency care where it may aid in the identification of patients at high risk of mortality who should be treated in an intensive care setting. There is little evidence in favor of suPAR guided algorithms in the management of specific complaints (chest or abdominal pain, dyspnea, fever) and at this point discharging patients on the basis of normal suPAR levels poses unacceptable risk. suPAR measurements could be more smoothly integrated in mainstream clinical practice if utilized in combination with other established prognostic and diagnostic biomarkers of sepsis. It should however be emphasized that laboratory tests or biomarker-guided algorithms are best utilized as an aid to management decisions which are first and foremost derived from a clinical evaluation of the patient. Evidence-based management algorithms and guidelines based on biomarkers may facilitate more effective management decision-making but they are not a substitute for clinical judgment. This is especially true for initial evaluation in the setting of emergency department.
